# *Leishmania* (*Viannia*) *naiffi* Lainson & Shaw 1989

**DOI:** 10.1186/s13071-023-05814-0

**Published:** 2023-06-08

**Authors:** Lilian Motta Cantanhêde, Elisa Cupolillo

**Affiliations:** 1 Leishmaniasis Research Laboratory, Oswaldo Cruz Institute, Fiocruz, Rio de Janeiro, Brazil; 2 Instituto Nacional de Ciência e Tecnologia de Epidemiologia da Amazônia Ocidental, INCT EpiAmO, Porto Velho, Brazil

**Keywords:** *Leishmania* (*Viannia*) *naiffi*, Epidemiology, Clinical outcomes

## Abstract

Just over 30 years ago, a new species of *Leishmania* of the subgenus *Leishmania* (*Viannia*) was described infecting the armadillo *Dasypus novemcinctus*; then, a report of human infection followed. From the Brazilian Amazon and apparently restricted to this region and its close borders, *Leishmania* (*Viannia*) *naiffi* has been characterized as a species that grows easily in axenic culture medium and causes few to no lesions after inoculation in experimental animal models. Results in the last decade indicate the occurrence of *L*. *naiffi* in vectors and human infections, including a report of therapeutic failure possibly associated with *Leishmania* RNA virus 1. Overall, such accounts suggest that the parasite is more dispersed and the disease less self-healing than previously expected.

**Graphical Abstract:**

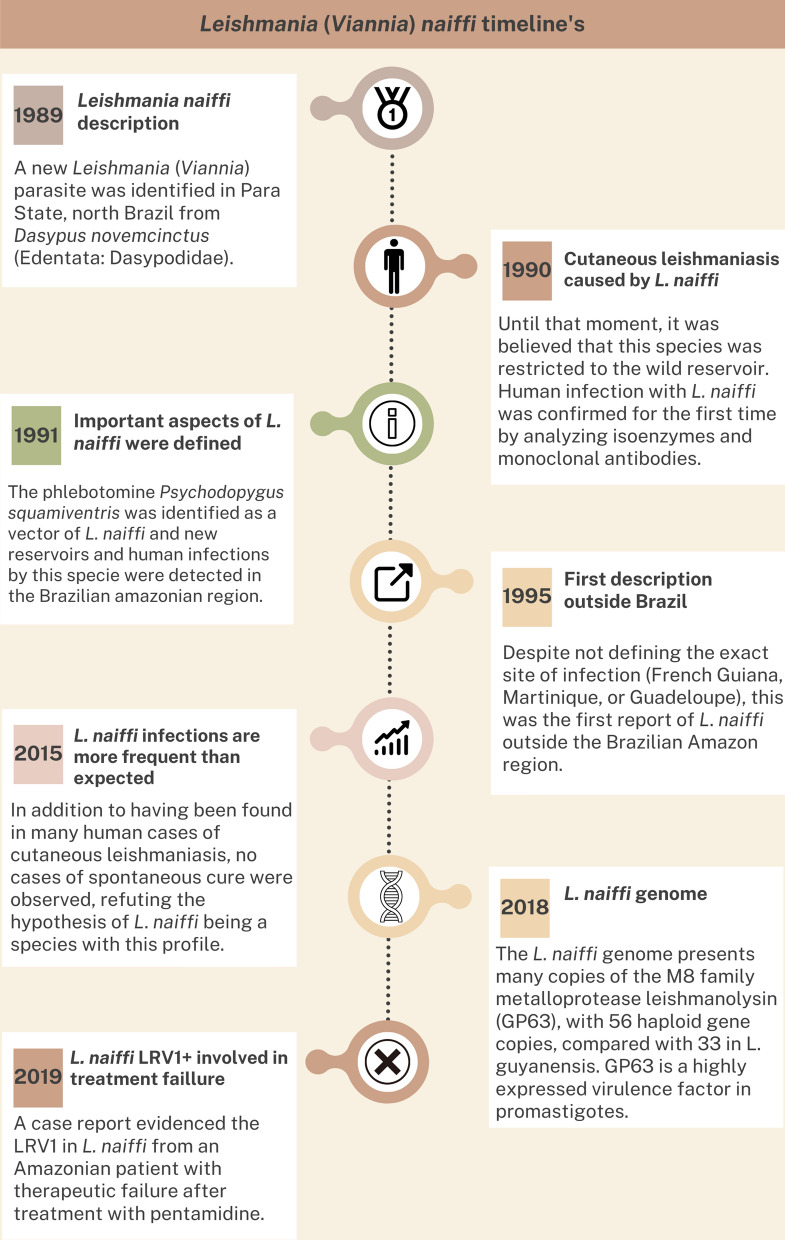

## Introduction

**Fig. 1:**
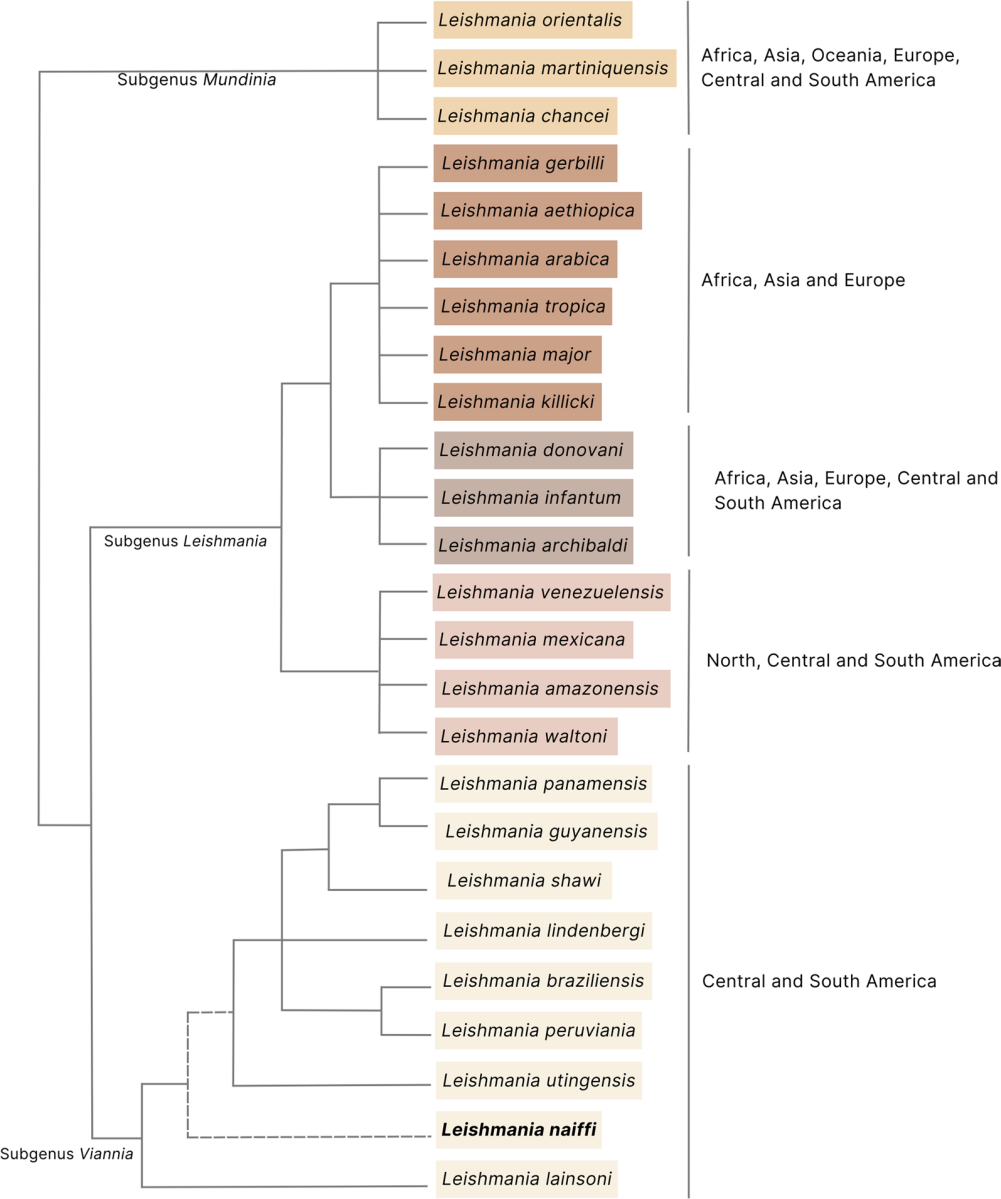
Representative phylogenetic tree of all 25 *Leishmania* species pathogenic to humans and classified in the subgenera *Mundinia*, *Leishmania* and *Viannia*. On the right, the indication of the continents where species of each subgenus have already been reported. *Leishmania naiffi* is highlighted. All *Leishmania* reference descriptions were revised by Das Chagas et al. [^1^], with the exception of *Leishmania* *chancei* [^2^]

The genus *Leishmania* includes 40 species classified by some subgenera [1], three comprising human pathogens: (i) *Leishmania* (*Mundinia*), a newly described group including six species, three associated with visceral (VL) and/or cutaneous leishmaniasis (CL), distributed worldwide [2]; (ii) *Leishmania* (*Leishmania*), comprising some species exclusively found in regions of Africa, Asia and Europe, but others in American regions, most of them human pathogens that cause CL, but also species that cause VL, named *Leishmania* *donovani* complex, one of which, *L*. (*L*.) *infantum*, is found in all these regions [3]; and (iii) *Leishmania* (*Viannia*), with nine species found in the Central and South American regions, all but one being human pathogens associated with CL. The *Leishmania* (*Viannia*) subgenus comprises two groups of related species, one including *Leishmania* (*V*.) *braziliensis* and *L*. (*V*.) *peruviana*, another including *L*. (*V*.) *guyanensis*, *L*. (*V*.) *panamensis* and *L*. (*V*.) *shawi*. Few studies were conducted on *Leishmania* (*V*.) *lindenbergi* and *L*. (*V*.) *utingensis*, but some point to *L*. (*V*.) *lainsoni* and *L*. (*V*.) *naiffi* as very distinct species classified in this subgenus [4] (Fig. 1).

*Leishmania* (*Viannia*) *naiffi* was considered a common parasite of the armadillo *Dasypus novemcinctus*, circulating in the Brazilian Amazon region. First classified as an unnamed member of the subgenus *Viannia*, *L. naiffi* was finally named in 1989 and typed in the subgenus *Viannia* based on biochemical and immunological characteristics [1–3]. In 1990, the first case of human cutaneous leishmaniasis (CL) caused by *L. naiffi* was described, but by then the etiology in many of the human patients infected with *L. naiffi* had probably been concealed by the *L*. (*Viannia*) spp. definition as a consequence of the inapparent type of infection produced in the skin of hamsters [5]. Notably, in the past, the inoculation of macerates of biopsies from skin lesions in hamsters was a method primarily employed to improve the success of parasite isolation.

The pioneering study described *L*. *naiffi* as a new *L*. (*Viannia*) species observed in 17 infected armadillos, showing high rates of parasite isolation in culture from spleen samples. All attempts to isolate the parasite from skin samples (from the ears and nose) failed, and the authors characterized *L*. *naiffi* as an essentially visceralizing parasite [6]. Hamsters infected by* L*. *naiffi* presented few amastigotes at the site of inoculation in the skin, producing inapparent infections or very discrete nodules [5].

**Fig. 2:**
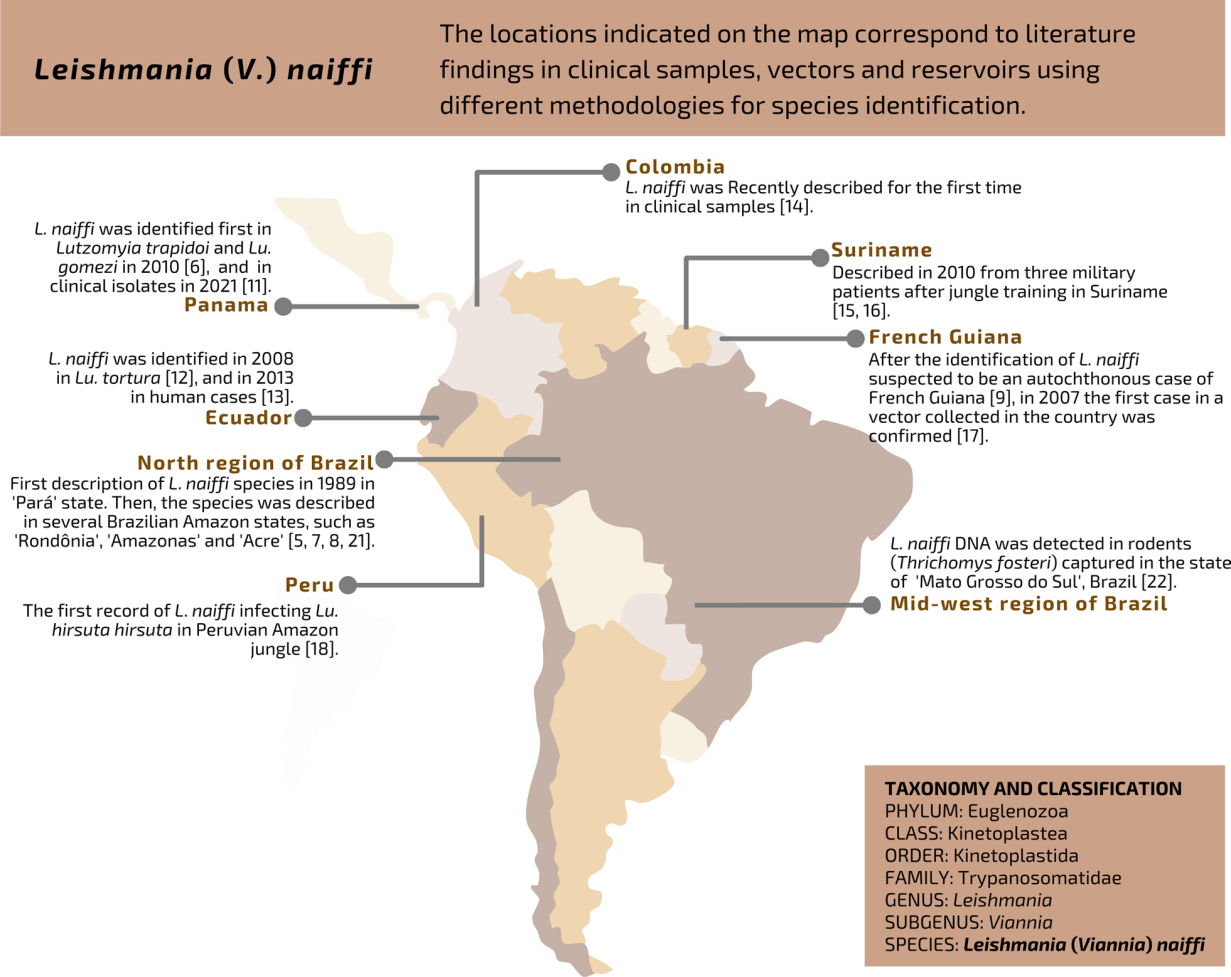
Geographic distribution of *Leishmania* (*Viannia*) *naiffi*

Since *L. naiffi’s* description and characterization, reports of infections with this species have remained infrequent in the literature. Little knowledge about its distribution has accumulated, mainly due to its overlap with other species of the subgenus *Viannia* [7, 8]. This is still a problem in the identification of species of this subgenus. In 1995, the first known case of infection with *L*. *naiffi* outside Brazil was reported [9]. Such identification of human infections with the parasite in neighboring countries had been expected because its probable vectors and reservoirs are widely distributed throughout most of South America. In later years, human and insect infections were recorded in Panama [10, 11], Ecuador [12, 13], Colombia [14], Suriname [15, 16], French Guiana [17] and Peru [18] (Fig. 2).

### Vectors

**Fig. 3:**
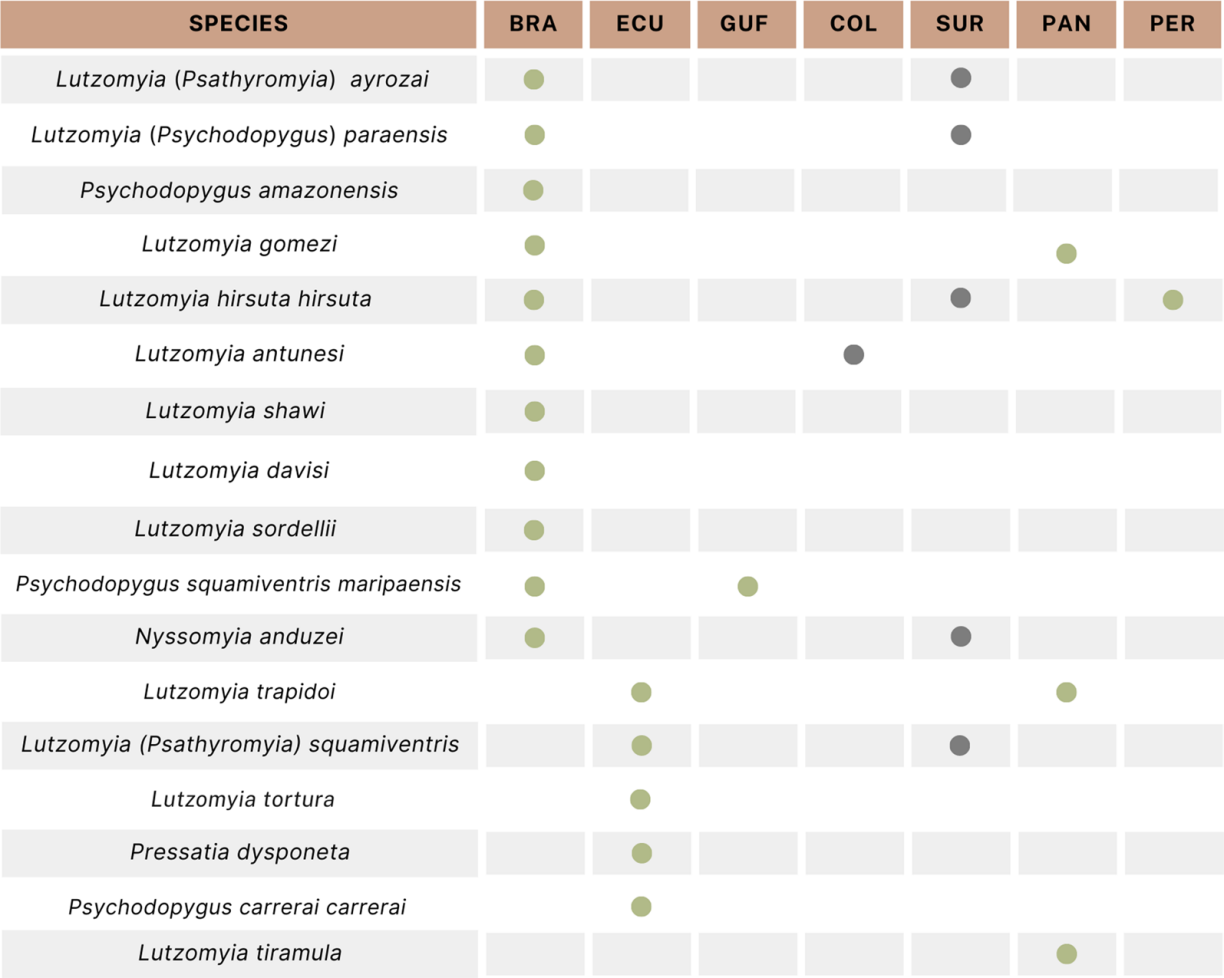
Sandfly species suspected or incriminated as vectors of *Leishmania naiffi* in countries where this parasite circulates. *BRA* Brazil, *ECU* Ecuador, *GUF* French Guiana, *COL* Colombia, *SUR* Suriname, *PAN* Panama, *PER* Peru. The green dots represent vectors detected with *L*. *naiffi*. The gray dots show countries where the parasite circulates, but it was not detected in insects already identified in the region

Many sandfly species are suspected or incriminated as vectors of *L*. *naiffi* (Fig. 3), including *Lutzomyia* (*Psathyromyia*) *ayrozai, Lu*. (*psychodopygus*) *paraensis, Psychodopygus amazonensis* and *Lu. gomezi* in Brazil [19, 20]; *Lu*. (*Psathyromyia*) *squamiventris* and *Lu*. *tortura* in Ecuador [12]; and *Lu*. *trapidoi* and *Lu*. *gomezi* in Panama [10]. This broad distribution of implicated sand flies enforces speculation that the dispersion and frequency of *L. naiffi* are more significant than what has been reported in the literature [10, 12, 13, 17, 21–25].

*Leishmania* *naiffi* vectors have distinct behavior profiles. For example, while *Lu*. (*Psathyomyia*) *squamiventris* is highly anthropophilic, *Lu*. (*Psathyromyia*) *ayrozai* does not share that behavior [5, 26]. This allows the circulation of the parasite in environments with different levels of anthropotization.

While in some regions, like Brazil, the sand fly fauna has been widely studied and consequently its diversity and the association of some of its species with *L. naiffi* transmission have been established, in others, such as Suriname and Colombia, the vectors or probable vectors of *L*. *naiffi* are still elusive. Nevertheless, sand flies implicated in the transmission of the parasite elsewhere can be found in these countries. For example, *Lutzomyia* (*Nyssomyia*) *anduzei*, *Lutzomyia* (*Psychodopygus*) *ayrozai*, *Lutzomyia* (*Psy*.) *hirsuta hirsuta* and *Lutzomyia* (*Psy*.) *squamiventris* can be found in Suriname [27], while *Lu*. *antunesi* is part of the fauna in Colombia [28]. This information gap needs to be closed to enrich epidemiological measures.

In a recent study, *L*. *naiffi* was the most frequent species of *Leishmania* detected in phlebotomine sandflies collected in Porto Velho, Northern Brazil. It was present in almost 10% of the analyzed pools of *Lu*. *antunesi*, *Lu*. *davisi*, *Lu*. *hirsuta hirsuta*, *Lu*. *shawi*, *Lu*. *sordellii* and *Lu*. (*Trichophoromyia*) spp. [29]. Interestingly, *L*. *naiffi* has not yet been reportedly detected in human cases of leishmaniasis in that region [30, 31]. Thereafter, *L*. *naiffi* was described for the first time in *Psychodopygus amazonensis* and *Lu. gomezi* from another Brazilian Amazon region [32].

The first description of a potential sand fly infection with *L. naiffi* in Peru consisted of the detection of *L*. *naiffi* DNA in a nucleic acid extract from a *Lu*. *hirsuta hirsuta* pool [18]. The insects had been collected in San Martin, which is not located close to Ecuador or Acre, in Brazil, where infections of sand flies by *L*. *naiffi* have been reported. Altogether, these recent results indicate the underestimated relevance and dispersion of *L*. *naiffi* in the Amazon region.

### Reservoirs

**Fig. 4:**
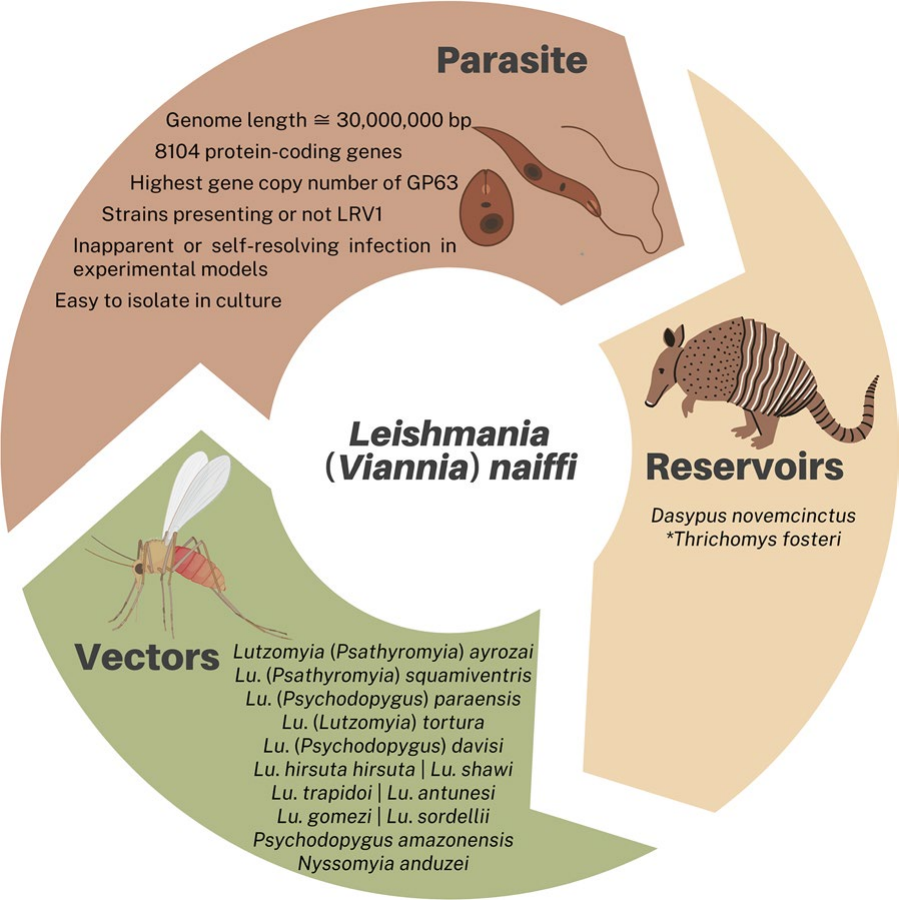
*Leishmania* (*Viannia*) *naiffi* characteristics, its main vectors and reservoirs. *L. *naiffi* DNA detection

The main reservoir of *L*. *naiffi* is the *D. novemcinctus* [6, 33], a nine-banded armadillo considered a pest but commonly consumed in the Americas [34–36] (Fig. 4).

In the Brazilian Amazon region, the armadillo has been considered the fundamental source of *Leishmania* spp. parasites for many years because of the frequency of sandflies in armadillo burrows. Armadillo in Indian Tupi is 'Tatu,' while the sand fly is 'tatuquira,' like the armadillo fly, a popular Brazilian name given to the insect vector of leishmaniasis [37]. Although *D. novemcinctus* is probably the principal reservoir of *L*. *naiffi*, this parasite was detected in the caviomorph rodent *Thrichomys fosteri* in Mato Grosso do Sul, central western Brazil [38]. This opens a venue for investigating the possibility of other mammals participating in the transmission cycle of *L*. *naiffi* and raising the alarm about a more significant geographic distribution of this species in areas outside the Amazon, although apparently with a transmission cycle that still does not involve humans.

### Parasite characteristics and infection profile in humans and experimental animals

Despite little knowledge about the distribution of this species, some characteristics of the parasite are intriguing, such as its exuberant growth in culture medium versus low replication after animal inoculation [6, 37], and the highest number of copies of the M8 gene, metalloprotease leishmanolysin family (GP63) [39] versus the correlation of benign course of *L*. *naiffi* infection [15]. A putative hybrid between *L. naiffi* and *L. lainsoni*, the most divergent species classified in the subgenus *Viannia* [40], was isolated from a female living in the Brazilian Amazon region (Acre State) presenting a cutaneous lesion [41].

Another mind-boggling feature is the presence of *Leishmania* RNA virus 1 (LRV1) in *L. naiffi*. Considering 18 *L*. *naiffi* strains available at the Leishmania Collection in Fiocruz, 13 are LRV1 positive (data available at clioc.fiocruz.br). Although the most commonly reported *L*. *naiffi* infections present benign courses [5, 16, 33], some studies paradoxically show non-self-healing infections, including a case report of treatment failure observed in a patient infected by an LRV1-positive *L*. *naiffi* strain [42, 43].

Some other *L*. *naiffi* aspects also seem paradoxical. The growth of cultured promastigotes is exuberant as GP63 levels are more expressed in *L*. *naiffi* promastigotes than in those of other species considered more pathogenic. GP63 levels have been correlated with increased *Leishmania* spp. virulence, and this surface molecule has been shown important for parasite entry into macrophages [44, 45]. So, overproduction of GP63 within a potentially promastigote-rich inoculum seems in disagreement with the apparently benign course of the disease and the scarcity of reported human *L*. *naiffi* infections.

Like in other *L*. (*Viannia*) species, an NADH-dependent fumarate reductase gene was amplified in *L*. *naiffi*, with 16 copies reported [39]. This gene is related to parasite resistance to oxidative stress, which potentially aids in persistence, drug resistance and metastasis. In comparative experimental infections with *L. braziliensis* and *L. lainsoni*, better control of the disease was associated with *L. naiffi*, especially in the earlier phase of infection, with low parasite numbers in paws and normal paw volumes and no parasites detection in lymph nodes at late points [46]. Another comparative study showed that the *L*. *naiffi* infection index was significantly lower than that in a *L*. *braziliensis* model with the NO production being higher, showing a negative correlation between these aspects [47]. *Leishmania naiffi* also demonstrated surprisingly longer survival times inside murine macrophages [48].

The visceralizing profile associated with *L*. *naiffi* leads to the hypothesis that this species could be more related to cases of mucosal leishmaniasis. However, properly addressing this hypothesis faces the hurdles imposed by the frequent negligence in screening this often chronic and insidious clinical outcome and the fact that routine *Leishmania* species identification still is not widespread in the foci of leishmaniasis transmission in the Americas.

It is also important to point out that the observation of mild to subclinical infections in humans and experimental animals, accompanied by the detection of viable parasites in their specimens, seems to suggest that *L. naiffi* may be silently maintained, possibly for a prolonged time, in the infected body.

All these discreet findings on *L*. *naiffi* show a widely dispersed parasite, especially in the Amazon, an area under increasing exploitation. *Leishmania* *naiffi* has also been reported in a Brazilian extra-Amazonian area with the expansion of human cases of CL and VL [49], which may represent a potential future spread. Furthermore, *L*. *naiffi* is likely more dispersed than what we know, considering the difficulty in identifying species of the subgenus *Viannia* and the diversity of vectors that have already been identified with DNA from *L*. *naiffi*. It is important to emphasize that the benign profile associated with infection by *L*. *naiffi* also may be related to the few reports in the literature. The poor immunological approaches to *L*. *naiffi* show a parasite that causes little 'noise' in the immune system from the initial moments of infection until its establishment, with a fitness prepared to respond to the main defense mechanisms of the host. It remains to be seen whether this strategy is reflected in more successful infections.

## Data Availability

Not applicable.
