# Correction: *Leishmania* (*Viannia*) *naiffi* Lainson & Shaw 1989

**DOI:** 10.1186/s13071-024-06352-z

**Published:** 2024-06-24

**Authors:** Lilian Motta Cantanhêde, Elisa Cupolillo

**Affiliations:** 1 Leishmaniasis Research Laboratory, Oswaldo Cruz Institute, Fiocruz, Rio de Janeiro, Brazil; 2 Instituto Nacional de Ciência e Tecnologia de Epidemiologia da Amazônia Ocidental, INCT EpiAmO, Porto Velho, Brazil

**Correction: Parasites & Vectors (2023) 16:194** 10.1186/s13071-023-05814-0

Following publication of the original article [1], it came to the journal's attention that the images of Figs. 3 and 4 had been erroneously swapped. The figures have since been corrected. The authors thank you for reading this erratum and apologize for any inconvenience caused.
